# Effects of Sitting Baduanjin on Cancer-Related Fatigue in Patients With Advanced Cancer: Protocol for a Randomized Controlled Trial

**DOI:** 10.2196/84925

**Published:** 2026-04-07

**Authors:** Qinghui Zhang, Xi Chen, Yinglong Duan, Jianfei Xie, Yongyi Chen, Yonghong Hu, Qinqin Cheng

**Affiliations:** 1 Hunan Cancer Hospital, No. 283, Yuelu District, Changsha, Hunan, China, +86-0731-89762400; 2 The Hong Kong Polytechnic University, Hong Kong, China; 3 The Third Xiangya Hospital, Central South University, Changsha, 410013, China

**Keywords:** sitting Baduanjin, patients with advanced cancer, cancer-related fatigue, quality of life, study protocol

## Abstract

**Background:**

Approximately 60% of patients with advanced cancer experience the distress of cancer-related fatigue (CRF), which significantly worsens their daily function and quality of life. Baduanjin has been regarded as a promising nonpharmacological intervention for alleviating CRF, anxiety, and depression and improving quality of life. Owing to varying degrees of CRF in patients with advanced cancer, patients may have insufficient endurance, making it difficult to implement standing Baduanjin. However, relevant evidence on sitting Baduanjin for CRF in patients with advanced cancer is lacking.

**Objective:**

This study aims to design a sitting Baduanjin intervention and explore the efficacy and safety of sitting Baduanjin in reducing CRF among patients with advanced cancer.

**Methods:**

This study will be a single-blind pilot randomized controlled trial. Patients with CRF will be enlisted from a tertiary cancer hospital in China. The participants (N=98) will be randomly assigned to either a sitting Baduanjin group or a control group at a 1:1 ratio using a block-randomized scheme. The participants in the sitting Baduanjin group will undergo a 16-week sitting Baduanjin intervention in addition to standard care, whereas the participants in the control group will receive standard care in the form of a booklet on the self-management of cancer symptoms. CRF will constitute the primary outcome, whereas anxiety, depression, and quality of life will serve as the secondary outcomes. These outcomes will be assessed at baseline (T0), 12 weeks (T1), and 16 weeks (T2).

**Results:**

The study was approved by the Medical Ethics Review Committee of Hunan Cancer Hospital in March 2024 (ethics approval number 2024.50). Before this full-scale randomized controlled trial, we conducted a pilot study, which demonstrated the feasibility and acceptability of sitting Baduanjin for patients with advanced cancer, with potential benefits for relieving fatigue.

**Conclusions:**

This randomized trial will evaluate the effectiveness of sitting Baduanjin exercises in alleviating CRF among patients with advanced cancer. If proven effective, it will provide a promising alternative intervention for patients with advanced cancer.

## Introduction

Cancer-related fatigue (CRF) is a distressing, lasting, subjective state of physical, emotional, and/or cognitive tiredness that is not in proportion to recent activities. It is related to cancer or associated treatments and is frequently accompanied by dysfunction [1]. Approximately 60% of patients in the advanced stage of cancer experience CRF [2]. CRF is one of the most prevalent symptoms manifested by patients with advanced cancer, and it can considerably deteriorate their quality of life and daily functioning [3]. Furthermore, it constitutes one of the primary reasons for the disruption of treatment or the deterioration of the disease [4]. Therefore, relieving CRF for patients with advanced cancer is critical.

At present, there is no acknowledged standard for the treatment of CRF [5] as its pathogenesis remains unclear. It could be associated with proinflammatory cytokines, growth factors, modulation of the circadian rhythm, disruption of the adrenal axis, serotonin imbalance, afferent activation of the vagus nerve, or the generation or use of abnormal adenosine triphosphate [6]. Previous research has shown that aerobic exercise (including tai chi and yoga) might be an effective CRF intervention and prove to be more efficacious than existing pharmaceutical treatments in alleviating CRF [7,8]. The current guidelines for the treatment of CRF recommend exercise-based activities rather than pharmaceutical interventions [9]. Aerobic exercise mitigates CRF both during and after treatment, particularly for those with solid tumors [10], and contributes to alleviating the cognitive and physiological consequences of fatigue [11]. Despite the existing evidence on the positive effects of aerobic exercise, many patients with advanced cancer cannot adhere to or perform exercise while standing due to weakness [12]. Therefore, it is necessary to find an appropriate exercise for those patients with advanced cancer who need to exercise while sitting.

Baduanjin, a traditional Chinese mind-body workout consisting of just 8 simple movements, is a proper moderate-intensity exercise for CRF in the treatment of advanced cancer as it is easy to master, consumes less energy, and requires no specific equipment [13]. Previous research indicates that Baduanjin is capable of alleviating the fatigue symptoms of patients with diverse diseases [14,15]. From the viewpoint of traditional Chinese medicine, CRF belongs to the category of deficiency and is marked by insufficient visceral functions and deficiencies in *qi*, blood, *yin*, and *yang*. Baduanjin can relieve CRF by facilitating the smooth flow of meridians, enhancing *qi*, improving blood circulation, regulating internal organ functions, boosting the immune system, and eliminating harmful toxins [16,17].

However, the current research on Baduanjin for patients with advanced cancer mainly focuses on standing Baduanjin. Due to varying degrees of CRF in patients with advanced cancer, patients may have insufficient endurance, making it difficult to implement standing Baduanjin. Therefore, sitting Baduanjin is more suitable for these patients. Currently, there is little research on sitting Baduanjin. One study developed a sitting Baduanjin intervention for patients with sepsis undergoing noninvasive ventilation [18]. The findings suggest that sitting Baduanjin could improve patients’ muscle strength and activities of daily living [18]. Another study developed a sitting Baduanjin intervention for older adults and found that it improved their balance and motor function [19]. Previously, the evidence on the benefits of Baduanjin mainly came from studies on patients with breast cancer and other populations. Before this trial, the efficacy of Baduanjin in relieving fatigue in patients with advanced cancer had not been confirmed. Due to the differences in symptom burden, there is currently no direct empirical verification of the impact of the previously developed sitting Baduanjin interventions on patients with advanced cancer. This emphasizes a significant need to investigate the effects of performing sitting Baduanjin on CRF in patients with advanced cancer.

## Methods

### Study Design

**Figure 1.**
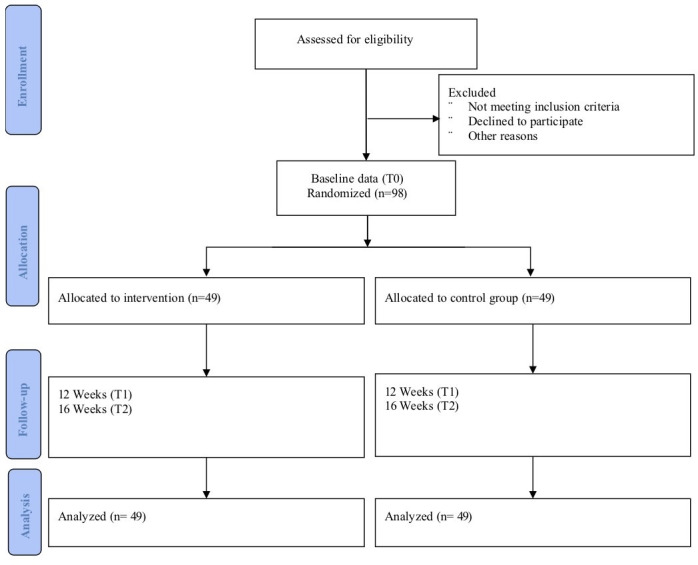
CONSORT (Consolidated Standards of Reporting Trials) flow diagram of the study.

This research is a single-blinded, parallel randomized controlled trial (RCT) with 2 arms. An overall diagram of the research is presented in Figure 1. The schedule of the study adheres to the SPIRIT (Standard Protocol Items: Recommendations for Interventional Trials) guidelines [20].

### Study Setting and Participants

Participants will be enlisted from a tertiary cancer hospital in Hunan, China. Patients with cancer will be eligible for enlistment if they meet the following criteria: (1) diagnosis of stage III or IV cancer; (2) age of >18 years; (3) being informed of their illness; (4) capability of communicating and understanding the research; (5) willingness and ability to provide written informed consent for study participation; and (6) Revised Piper Fatigue Scale (RPFS) score of ≥4, indicating moderate to severe fatigue. Patients will be excluded in case they meet any of the following exclusion criteria: (1) currently using medications for fatigue, sleep disturbance, or depression, such as antidepressants, psychostimulants, or hypnotics; (2) being too frail to participate in physical activities due to terminal stages of chronic illnesses or having cognitive impairment and/or severe mental illness; (3) having serious exercise contraindications, such as cardiovascular or joint problems; and (4) participating in other exercise research programs.

### Sample Size Calculation

The sample size was computed using the G*Power software (version 3.10). No previous research reports have emerged regarding the effect of sitting Baduanjin on CRF in patients with advanced cancer. Therefore, the effect value was set to 0.50. In the G*Power software, the *t* test for the mean difference between 2 independent samples was selected. With an effect size of 0.69 [21], a statistical power of 0.85, and a 2-tailed test, the sample size for each group was calculated to be 39. Thus, considering a conservative anticipation of a 20% dropout rate, the final sample size will be 49 in each group, with a total of 98 participants.

### Recruitment

Patients with advanced cancer will be approached and recruited in a tertiary cancer hospital by a research nurse. This research nurse will screen patients with advanced cancer according to the inclusion and exclusion criteria. Information on this study, encompassing the purpose, the intervention, and the possible benefits and harms, will then be offered to the potential participants to obtain their informed consent. In addition, recruitment posters will be displayed in the study hospital to expand recruitment. After the participants’ consent is obtained, the researchers will invite the participants to provide baseline data using paper questionnaires.

### Randomization and Allocation Concealment

The participants will be randomly allocated into 2 groups in a 1:1 ratio using a block-randomized scheme with block sizes of 4 or 6. One set of randomization sequences will be generated through an online randomizer [22] based on the estimated sample size. To guarantee allocation concealment, the randomization sequences will be produced by a research assistant who will not be involved in any other part of this study. The randomization sequences will then be put into sequential, sealed, opaque envelopes and kept by another research assistant who is not involved in the study. The randomization sequences will be inaccessible to other research staff. Once an eligible participant gives consent to take part in the study and finishes the baseline assessment, the research nurse in charge of recruitment will call the research assistant to disclose the allocation result.

### Blinding

Owing to the nature of the sitting Baduanjin intervention, it will be impossible to blind the study investigators and participants. Consequently, blinding will only be implemented for the outcome assessors in this RCT to prevent potential detection bias during data collection. The outcome assessors will not be involved in the participant recruitment process. To ensure objective and unbiased results, the people in charge of data collection and analysis will not be informed of the group details.

### Experimental Group

The participants in the experimental group will receive the sitting Baduanjin program. The sitting Baduanjin intervention was developed according to the characteristics of CRF under the guidance of the *zangfu* theory of traditional Chinese medicine and the *qi*-blood meridian theory. In addition, we improved the original standing Baduanjin workout by integrating relevant movements. This plan was revised through 2 rounds of expert consultation, resulting in the improved sitting Baduanjin intervention.

The sitting Baduanjin workout consists of 8 movements, which involve the upper limbs, head and neck, and spine, as well as organized breathing. A preparation and closing posture are added before and after the 8 movements to prepare the patient and ensure their safety [23]. The movements of the sitting Baduanjin workout were tested among patients with advanced cancer for preliminary feasibility and safety. The detailed movements of sitting Baduanjin are shown in Table 1.

**Table 1.** The movements of sitting Baduanjin.

Movement	Main points of the movement
Preparation posture	The patient sits on a chair with their legs naturally open or on the bed. With the body straight and facing the front, the patient feels calm, at ease, and levitated at the Baihui acupoint. The tongue is pressed against the upper jaw. The patient puts their palms in front of the abdomen, closes their eyes, and breathes slightly and peacefully.
First movement: “hands in the sky to support Sanjiao”	When inhaling, the patient lifts their hands from their chest to the top of their head. When exhaling, the patient drops their hands from both sides of their body to their waist and holds them in front of their abdomen.
Second movement: “drawing the bow to shoot the hawk”	The patient reaches their arms out to the sides with their palms down as if they were holding a bow and arrow. They inhale and then exhale as they draw their arms back, bending their elbows and bringing their hands toward their chest. They hold for a moment and then release by straightening their arms and returning to the starting position.
Third movement: “lifting the arms to regulate the spleen and stomach”	The patient raises the left palm and rotates it through the chest, then brings it upward in front of their face, followed by an inward rotation of the arms to lift it to the upper left side of the head. Simultaneously, they rotate the right palm inward and press it down beside the right hip with their fingertips facing forward. The patient then drops their left hand, lifts their right hand simultaneously, and holds their 2 hands in front of their abdomen. They repeat the same movement on the opposite side.
Fourth movement: “looking backward to relieve the fatigues and 7 injuries”	The patient rotates both arms outward with the palms facing outward, turns their head to the left and back, pauses for a moment, and looks diagonally behind to the left. They rotate both arms inward, press them against their hips, move their fingers forward, and look straight ahead. They repeat the same movement on the opposite side.
Fifth movement: “clearing the heart fire by shaking the head and wagging the tail”	The patient lifts both palms in front of the abdomen and chest, extending them above their head with their fingers facing each other. They lower their arms to both sides, placing their palms on top of their thighs. They raise the center of gravity slightly, then shift it to the right, tilt their upper body to the right, bend down, and visually observe the surface of their right foot. They shift the center of gravity to the left while rotating their upper body from right to front and left, visually observing their right heel. They shift the center of gravity to the right, shake their head backward, slightly retract their jaw, and look ahead. The right-side action is the same as the left-side action but in the opposite direction.
Sixth movement: “move 2 hands down to the leg to strengthen the kidney and lumbar area”	The patient raises both arms forward and upward, with their palms facing forward. They press their palms against their chest with the elbows bent, palms facing downward, and fingers facing each other. They rotate both arms outward with their palms facing upward and then insert the palm fingers under the armpits. They rub their palms inward along the spine to the kidney area and then rub them back and forth. They return their hands to the front of the abdomen through the body.
Seventh movement: “tighten fists to gather strength”	The patient holds both fists at the waist, with the thumb inside and the fist facing upward, and looks straight ahead. They inhale and hold their fists, exhale and clench their left fist with a furious gaze, keep their eyes wide open, grip firmly, inhale, and retract. They exhale and clench their right fist with a furious gaze, keep their eyes wide open, grip firmly, inhale, and retract.
Eighth movement: “rotate shoulders to enhance *yang* and *qi”*	The patient lowers both arms naturally, contracts the abdomen, and expands the chest. They inhale to lift their shoulders and rotate them inward and exhale to lower their shoulders. They inhale to lift their shoulders and rotate them outward and exhale to lower their shoulders.
Closing posture	The patient wipes their face, combs their hair, rubs their ears, and taps their left and right elbow creases.

The program lasts for a total of 16 weeks [12]. In the first week, the participants will attend a training session to learn about sitting Baduanjin by watching a training video, which explains the exercise methods and precautions of sitting Baduanjin. A trained research nurse will then guide the participants to complete sitting Baduanjin according to the video and adjust incorrect movements individually, if any. The training session will include no more than 8 patients. From week 2 to week 16, the participants will perform sitting Baduanjin exercises by themselves once daily for approximately 20 to 30 minutes, including 5 minutes of preparation posture, 12 minutes of sitting Baduanjin, and 5 minutes of closing posture.

To enhance participant compliance, we have established a WeChat group for the experimental group to provide remote supervision. Videos of the sitting Baduanjin exercises and relevant theoretical knowledge will be pushed to patients via WeChat. Participants in the experimental group are required to practice daily, take photos as proof, and upload them via a check-in mini program. This process serves to document their practice duration and facilitates self-monitoring. Their caregivers are also involved to encourage participants to persist in their exercises. During home care, the main responsibility of the caregiver is to remind the participants to do the Baduanjin exercises on time and submit proof of having done the exercises via WeChat. We conduct follow-up visits and phone calls with patients twice a week via WeChat video to inquire about factors that may hinder their activities and resolve them promptly. In addition, participants in the experimental group are requested not to discuss their practice with each other to minimize interference.

### Control Group

Patients randomized to the control group are provided with usual care. They will receive regular health education on exercise and a healthy lifestyle, including seated stretching and breathing exercises. After discharge, they will be provided with material on how to exercise. Participants will be asked to avoid any other traditional Chinese sports activities during the study, with reminders provided at every assessment interval. Considering ethical aspects, once the effectiveness of sitting Baduanjin is established, we will provide it for free to the control group if they are interested.

### Outcome Measurements

#### Primary Outcomes

The primary outcome of this RCT is CRF. The severity of CRF in the daily functioning of the participants will be measured by using the Chinese version of the RPFS. The original scale was developed by Piper [24] in 1989, with its revised Chinese version developed by So et al [25] in 2003. The questionnaire includes 22 items that assess the general level of fatigue using a visual analog scale with values from 0 to 10. It also assesses 4 facets of fatigue: severity, emotional, sensory, and cognitive. A score of 0 indicates no fatigue symptoms, mild fatigue is defined as scores between 1 and 3, moderate fatigue is defined as scores between 4 and 6, and severe fatigue is defined as scores between 7 and 10. The RPFS exhibits Cronbach α coefficients between 0.89 and 0.93 for each subscale and 0.91 for the overall scale [26].

#### Secondary Outcomes

The secondary outcomes include patients’ anxiety, depression, and quality of life.

##### Anxiety

Secondary outcomes encompass anxiety as gauged using the mean score on the 7-item Generalized Anxiety Disorder Scale (GAD-7) [27]. The GAD-7 was developed by Spitzer et al [28] in 2006 for measuring the level of anxiety in patients over the previous 2 weeks. The GAD-7 is a gold-standard measuring instrument for generalized anxiety disorder [27]. It is a rapid, user-friendly, compact, and self-executed screening and diagnostic instrument. The GAD-7 is computed by allocating scores of 0, 1, 2, and 3 to the response categories of “not at all,” “several days,” “more than half the days,” and “nearly every day,” respectively. The total score ranges from 0 to 21. Scores of 5, 10, and 15, respectively, denote cutoff points for mild, moderate, and severe anxiety. The Chinese version of the GAD-7 shows solid internal consistency reliability (Cronbach α ranging from 0.715 to 0.931), along with construct validity and convergent validity [29].

##### Depression

Depression will be gauged by means of the Patient Health Questionnaire–9 (PHQ-9) [30]. The PHQ-9 is a self-administered diagnostic tool for the severity of depression [30]. It is determined by attributing scores of 0, 1, 2, and 3 to the response categories of “not at all,” “several days,” “more than half the days,” and “nearly every day,” respectively. The overall score ranges between 0 and 27. Scores of 5, 10, 15, and 20 stand for the cutoff points for mild, moderate, moderately severe, and severe depression, respectively. The Chinese version of the PHQ-9 exhibits internal consistency reliability (with Cronbach α ranging from 0.729 to 0.891), along with construct validity and convergent validity [31].

##### Quality of Life

Quality of life will be evaluated using the Functional Assessment of Cancer Therapy–General (FACT-G) total score. The scale encompasses 27 items across 4 dimensions: physiological status, social or family status, and functional status, each containing 7 items, and emotional status, containing 6 items. Each item is scored from 0 to 4, with higher scores denoting better quality of life [32]. The FACT-G is accessible in a simplified Chinese version, and adequate psychometric properties have been reported among patients with advanced cancer. The Chinese version of the FACT-G exhibits excellent internal consistency reliability (with Cronbach α from 0.89 to 0.98), along with construct validity and convergent validity [33].

##### Other Measurements

In addition, patient demographics (including gender, age, educational background, marital status, employment status, and household income) and clinical characteristics (such as diagnosis, stage, course of disease, and current treatment) will be collected at baseline.

### Data Collection

The baseline assessments will be conducted by the research nurse who is in charge of participant recruitment and random assignment to groups. All participants will be requested to complete the RPFS, GAD-7, PHQ-9, FACT-G, and demographic data sheet on-site at baseline (T0). The reassessment of the outcomes will be conducted at 12 (T1) and 16 (T2) weeks after baseline. Follow-up data will be gathered via telephone by a research assistant who will be blinded to the participants’ group allocation. The schedule of study outcome assessments is outlined in Table 2.

**Table 2.** SPIRIT (Standard Protocol Items: Recommendations for Interventional Trials) diagram of enrollment, treatment, and assessments over the intervention period.

	Study period					
	Enrollment	Baseline assessment	Allocation	Intervention week 12 (T1)	Intervention week 16 (T2)	
Recruitment						
Eligibility screen	✓					
Informed consent	✓					
Randomization						
Allocation			✓			
Interventions						
Experimental group			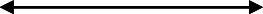			
Control group			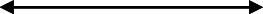			
Patient-reported outcomes						
Demographic characteristics		✓				
RPFS^a^		✓		✓	✓	
GAD-7^b^		✓		✓	✓	
PHQ-9^c^		✓		✓	✓	
FACT-G^d^		✓		✓	✓	
Adverse effects or events				✓	✓	
Adherence to exercise			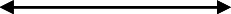			

a RPFS: Revised Piper Fatigue Scale.

b GAD-7: 7-item Generalized Anxiety Disorder Scale.

c PHQ-9: Patient Health Questionnaire–9.

d FACT-G: Functional Assessment of Cancer Therapy–General.

### Data Analysis

Statistical analyses will be carried out using SPSS Statistics for Windows (version 23.0; IBM Corp). Demographic data will be summed and presented using appropriate descriptive statistics, such as frequency distributions and means and SDs. The homogeneity of baseline characteristics between the 2 groups will be evaluated using the independent 2-tailed *t* test, chi-square test, or Wilcoxon rank sum test as appropriate. A generalized estimating equation (GEE) model will be implemented for repeated multivariate analysis between the 2 study groups regarding the total scores and domain scores on the RPFS, GAD-7, PHQ-9, and FACT-G. The analysis will be conducted in accordance with the intention-to-treat principle. In case of missing data at random, the missingness will be left unaddressed because the GEE model could yield valid results through analyzing the observed data [34]. Otherwise, multiple imputation will be used for the missing data. Sensitivity analyses will be conducted by comparing results across standard GEE and multiple imputation–based GEE. The significance level for identifying statistical differences will be set at *P*<.05.

### Ethical Considerations

The study will comply with the principles of the Declaration of Helsinki and the International Code of Ethics for Biomedical Research Involving Human Subjects. The study was approved by the Medical Ethics Review Committee of Hunan Cancer Hospital in March 2024 (ethics approval number 2024.50) and registered in the Chinese Clinical Trial Register (ChiCTR-2400092148). Written consent will be obtained from each participant before data collection. Participation in the study will not cause any harm to the patients. The patients will be notified that their participation is voluntary and that they have the right to withdraw from the study at any time without providing any explanation or facing any penalty. Data collection from the participants will adhere to the principles of confidentiality and anonymity. In addition, small gifts will be given to patients (eg, towels and toothbrushes worth approximately ¥20 [US $2.87]) to thank them for the time taken to fill out the questionnaires at each assessment. The records and all information gathered during the study will be stored in a locked cabinet. Electronic data will be stored in a password-protected storage device. Only the researchers will be granted access to the data, which will be merely used for research purposes. The data will be deleted 5 years after the completion of the study.

## Results

The study was approved by the Medical Ethics Review Committee of Hunan Cancer Hospital in March 2024 (ethics approval number 2024.50). Before this full-scale RCT, we conducted a pilot study. A total of 24 participants were recruited between March 2025 and April 2025. After the intervention, 18 participants, comprising 9 from the intervention group and 9 from the control group, completed the follow-up evaluation. The retention rate was 75%. The pilot study demonstrated the feasibility and acceptability of sitting Baduanjin for patients with advanced cancer, with potential benefits for relieving fatigue.

## Discussion

### Hypothesized Findings

Sitting Baduanjin helps control breathing, align the body, and soothe the mind while softly adjusting the functions of internal organs [35]. Therefore, it is supposed to relieve patients’ CRF, anxiety, and depression and improve their quality of life. As there is limited research on sitting Baduanjin, this study will contribute to the knowledge on sitting Baduanjin in traditional Chinese medicine. A meticulously designed RCT will be used to evaluate the impacts of the sitting Baduanjin program on alleviating CRF among patients with advanced cancer. If the effects of the sitting Baduanjin program are positive, this study will offer an appropriate Chinese traditional exercise approach for patients with advanced cancer who have insufficient endurance for standing exercises. In addition, the results will enrich the scientific and practical knowledge of interventions for relieving CRF among patients with advanced cancer.

Sitting Baduanjin’s effectiveness could be due to its unique features. First, the sitting Baduanjin program outlined in this protocol will be developed with consideration of the fatigue of patients with advanced cancer in accordance with the *zangfu* theory of traditional Chinese medicine and the *qi*-blood meridian theory. Second, patients with advanced cancer can easily access it. Sitting Baduanjin can be performed in a seated position, which suits their physical abilities and satisfies their exercise demands. Third, sitting Baduanjin involves meditation and deep breathing as a mind-body exercise, which might help decrease CRF [36]. Fourth, participants are encouraged by their caregivers to keep up with their exercises. Videos of sitting Baduanjin and theoretical knowledge were created to enhance patients’ adherence to exercise. This study will use daily exercise logs via an app to monitor home practice and record patients’ self-reported experiences with sitting Baduanjin. Furthermore, weekly conversations through WeChat or phone calls will be held to boost exercise self-efficacy.

This protocol has several potential limitations. First, because of the nature of the intervention, it will not be possible to blind the participants. This has the potential to introduce performance bias. Nevertheless, outcome assessors will be kept blind to minimize bias in the evaluation of the effects of the intervention. Second, due to the restricted study sites, the sample in this study might not be fully representative of patients with advanced cancer experiencing CRF. As patients will only be recruited from a tertiary cancer hospital, the results might not apply to all patients with advanced cancer.

### Future Directions

There are several strategies that can be used to enhance the reliability and comprehensiveness of the research on the effects of sitting Baduanjin in patients with advanced cancer experiencing CRF. First, it is crucial to increase the sample size and study duration by carrying out multicenter studies with more participants and longer intervention periods. Second, enhancing the research design by applying more rigorous randomization and blinding techniques is essential for reducing bias and guaranteeing the validity of the findings. Third, enhancing intervention strategies by integrating nutritional advice, psychological support, and other pertinent techniques can further boost the intervention’s effectiveness.

## Abbreviations

CRF: cancer-related fatigue
FACT-G: Functional Assessment of Cancer Therapy–General
GAD-7: 7-item Generalized Anxiety Disorder Scale
GEE: generalized estimating equation
PHQ-9: Patient Health Questionnaire–9
RCT: randomized controlled trial
RPFS: Revised Piper Fatigue Scale
SPIRIT: Standard Protocol Items: Recommendations for Interventional Trials
